# Ecological opportunity and the adaptive diversification of lineages

**DOI:** 10.1002/ece3.1347

**Published:** 2014-12-17

**Authors:** Gary A Wellborn, R Brian Langerhans

**Affiliations:** 1 Department of Biology, University of OklahomaNorman, Oklahoma, 73019; 2 Department of Biological Sciences and W.M. Keck Center for Behavioral Biology, North Carolina State UniversityCampus Box 7617, Raleigh, North Carolina, 27695

**Keywords:** Adaptive radiation, ecological community, ecotype, niche diversification, reproductive isolation, speciation

## Abstract

The tenet that ecological opportunity drives adaptive diversification has been central to theories of speciation since Darwin, yet no widely accepted definition or mechanistic framework for the concept currently exists. We propose a definition for ecological opportunity that provides an explicit mechanism for its action. In our formulation, ecological opportunity refers to environmental conditions that both permit the persistence of a lineage within a community, as well as generate divergent natural selection within that lineage. Thus, ecological opportunity arises from two fundamental elements: (1) niche availability, the ability of a population with a phenotype previously absent from a community to persist within that community and (2) niche discordance, the diversifying selection generated by the adaptive mismatch between a population's niche-related traits and the newly encountered ecological conditions. Evolutionary response to ecological opportunity is primarily governed by (1) spatiotemporal structure of ecological opportunity, which influences dynamics of selection and development of reproductive isolation and (2) diversification potential, the biological properties of a lineage that determine its capacity to diversify. Diversification under ecological opportunity proceeds as an increase in niche breadth, development of intraspecific ecotypes, speciation, and additional cycles of diversification that may themselves be triggered by speciation. Extensive ecological opportunity may exist in depauperate communities, but it is unclear whether ecological opportunity abates in species-rich communities. Because ecological opportunity should generally increase during times of rapid and multifarious environmental change, human activities may currently be generating elevated ecological opportunity – but so far little work has directly addressed this topic. Our framework highlights the need for greater synthesis of community ecology and evolutionary biology, unifying the four major components of the concept of ecological opportunity.

“Nothing in evolutionary biology makes sense except in the light of ecology”

Grant and Grant (2008, p. 167)

## Introduction

Ecological opportunity underlies adaptive diversification of species and may represent the primary environmental driver of phenotypic evolution, determining the rate and magnitude of lineage radiations. Despite the central place of the concept and its antecedents in historical (Darwin 1859; Simpson 1944) and current (Losos and Mahler 2010; Schluter 2000) theories of the development of biological diversity, the term “ecological opportunity” is used with a variety of definitions and descriptions, many very broad, and as a result the term has ambiguity in its meaning and a lack of precision in its mechanism of action. Here, we consider a mechanistic foundation for ecological opportunity that is grounded in its historical interpretation as an environmental setting conducive to adaptive diversification and to discuss current knowledge of the scope and action of ecological opportunity.

While we currently have no specific, widely accepted definition of “ecological opportunity,” common themes in its usage are evident. These themes pervade early exposition of the general concept by George G. Simpson and David Lack, who are often credited with the concept's inception. Simpson (1944) wrote “The availability of a new adaptive zone does not depend alone on its physical existence…, but also on its being open to other occupants (i.e., empty) or so sparsely or marginally occupied that it involves no great competition.” Lack (1947), perhaps without having yet read Simpson's book, inferred of the Galapagos' radiation of finches that “ancestors of Darwin's finches entered a lard of abundant food and varied living quarters, unmarred by the presence of competitive neighbours.” These formative treatments highlight features commonly ascribed to ecological opportunity: availability of empty and varied niches, underutilized resources, and the implication that these conditions underlie development of new biological diversity. These treatments also embraced the insight that ecological opportunity is prospective, as its name implies, and therefore may exist in a community even if an appropriate focal population has not yet encountered it. Recent usage of the term usually maintains these elements, with ecological opportunity defined, for example, as “the wealth of different resource types underutilized by species of other taxa” (Schluter 2000). Whereas these definitions focus on qualities of the environment itself, some authors offer definitions focused on the environment's impacts on a population or a population's response to the environment, as in “the relaxation of selection acting on some ecologically important trait” (Yoder et al. 2010). Similarly, ecological opportunity is often defined by stating predictions of what is commonly referred to as the “ecological opportunity hypothesis.” For example, the “ecological opportunity hypothesis proposes that organisms freed from the burden of competition … will experience a “release” characterized by bursts of phenotypic or morphological evolution and/or cladogenesis” (Burbrink and Pyron 2010). Researchers also regularly apply the concept to a wide array of scales, ranging from development of intraspecific polymorphism to diversification within a genus to the rise of mammal diversity following mass extinction (e.g., Parent and Crespi 2009; Pfennig and Pfennig 2012). Although these and other definitions capture substantive aspects of ecological opportunity, we feel a more elemental development of the concept is needed to yield greater clarity and provide an avenue toward greater utility.

We develop a mechanistic definition for ecological opportunity; one in which we restrict its meaning to environmental conditions that, when encountered by a lineage, directly cause divergent selection, and in which speciation, when it occurs under such conditions, produces ecologically diversified species. This emphasis on ecological opportunity's action as the driver of adaptive diversification follows the historical utility of the concept. Darwin (1859) saw ecologically mediated adaptive divergence as the mechanism of species formation, albeit without an appreciation of the genetic complexities involved. Simpson (1944, p. 200) viewed the process of speciation in explicitly adaptive and ecological terms: *“*the adaptive factor [in speciation] is adjustment to…differences in local ecological conditions,” as did Dobzhansky (1951, p. 9): *“*the enormous diversity of organisms may be envisaged as correlated with the immense variety of environments and ecological niches which exist on earth,” and Clausen (1951) emphasized adaptive ecological divergence as a key stage in plant speciation. Consistent with these formative ideas, our delimitation of the concept here, with its restricted scope, captures how ecological opportunity provides the circumstances under which adaptive diversification of species can occur.

**Figure 1:**
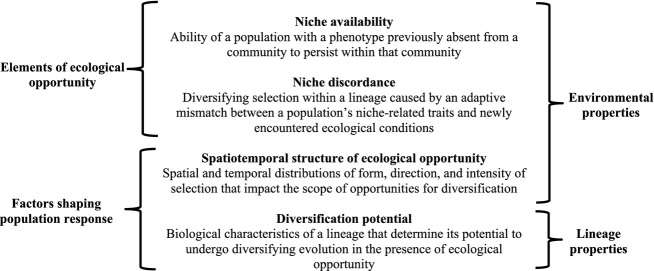
Mechanistic framework for structure and action of ecological opportunity. The fundamental constituents of ecological opportunity for a focal lineage are niche availability and niche discordance, which together generate the ecological substrate for diversifying evolution. Ecological opportunity occurs when environmental conditions allow both niche availability and niche discordance. Responses of lineages to ecological opportunity are shaped by its spatiotemporal structure and by lineage-specific biological properties, termed diversification potential.

Our framework organizes four fundamental elements through which ecological opportunity shapes adaptive diversification of lineages into multiple forms (Fig.1). Clearly, the underlying fabric of ecological opportunity is spatial and temporal heterogeneity of the ecological landscape, and it is from this variegated environmental setting that ecological diversification of species emerges. Ecological opportunity itself comprises two environmental constituents that may be experienced by a focal lineage: *niche availability* and *niche discordance*. Ecological opportunity is prospective, as it refers to conditions of an environment that a focal lineage may experience, but has not yet experienced. A lineage can experience ecological opportunity through colonization of a new location or an environmental change within its current location. A population's response to ecological opportunity is shaped by two major factors: the *spatiotemporal structure of ecological opportunity* and the population's *diversification potential*. Under appropriate circumstances, these four elements act in concert to yield ecologically driven lineage diversification.

## Niche Availability and Niche Discordance

Ecological opportunity exists only when environmental conditions permit the combination of two distinct elements should a lineage experience the environment. First, niche availability enables a focal lineage to survive and reproduce in the environment (Box 1). Second, niche discordance precipitates diversifying selection in the lineage due to altered ecological conditions (Box 2). Stated concisely, ecological opportunity refers to a prospective environment that, if encountered by a focal lineage, allows the lineage to persist (“niche availability”) while experiencing diversifying selection (“niche discordance”).

**Box 1. Niche Availability**

Establishment of a new phenotype within a community requires that a population maintain a viable population size in the face of both abiotic conditions and interspecific interactions experienced within the community. We use the term “new phenotype” to refer to a population possessing a phenotype not currently present in the community. Niche availability refers to the ability of a population with a phenotype previously absent from a community to persist within that community. More formally, niche availability can be characterized in two ways to aid in its conceptualization, empirical measurement, and theoretical applications: (1) zero net growth isoclines (ZNGIs) and impact vectors of mechanistic niche models (Chase and Leibold 2003) and (2) phenotypic adaptive landscapes of evolutionary biology (Simpson 1944). The two approaches differ in perspective, with the former (ecological) approach most useful for identifying environmental conditions conducive for niche availability for particular new phenotypes, and the latter (evolutionary) approach most helpful in identifying potential new phenotypes for which niche availability exists within given environments (Fig.4). First, ZNGIs are determined by the population dynamic response of a population to limiting environmental variables such as levels of key resources and density of predators, and impact vectors describe the impact of the population on dynamics of these environmental factors. Niche availability exists when environmental parameters allow a population with a new phenotype of some specific form to invade the community (Fig.4A; Holt et al. 1994; Chase and Leibold 2003; McPeek 2012). This model framework provides a tool for exploring factors shaping niche availability across different model communities, and parameters of these niche models are sometimes operationalized for laboratory and field studies (Chase and Leibold 2003), suggesting the possibility of their empirical application in the study of ecological opportunity. Second, niche availability can be described as a minimum mean population fitness (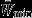) required for a population with a particular phenotypic distribution to maintain a viable population size and avoid extinction within a given environment (Fig.4B). Greater niche availability is described by broader or more numerous mean phenotypic values, not currently present within a community, having expected mean fitness at or exceeding 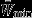.

**Figure I:**
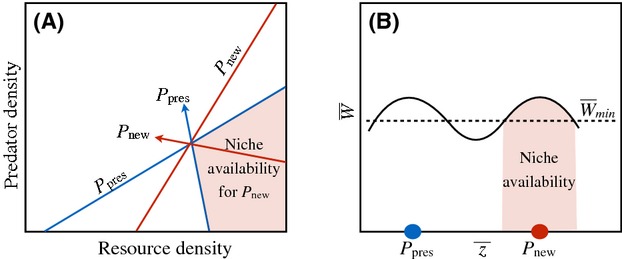
Two perspectives of niche availability for a population with a new phenotype previously absent within a community (*P*_new_) relative to a phenotype already present within the community (*P*_pres_). (A) Zero net growth isoclines (ZNGIs; lines) and impact vectors (arrows) for two populations with different mean phenotypic values that compete for a common resource and are consumed by a common predator. Niche availability for *P*_new_ exists only for environmental conditions found within the shaded region, with a stable equilibrium of coexistence where the ZNGIs intersect. (B) Adaptive landscape depicting mean population fitness across a range of mean phenotypic values encompassing both *P*_pres_ and *P*_new_, within an environment represented by the lower-right region of A where coexistence is possible. Populations with mean fitness ≥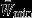 can maintain a viable population size. Niche availability within this environment exists only for new phenotypes within the shaded region.

**Box 2. Niche Discordance**

Niche discordance refers to diversifying selection generated by an adaptive mismatch between a focal population's niche-related traits and the environment's ecological conditions. In this context, “diversifying” selection describes selection for increased phenotypic variance within a lineage (or between newly diverging lineages), and may occur by a broadening of the selective surface (niche expansion) or disruptive selection within a population, or by divergent selection acting between spatially segregated subpopulations. Within a single community, niche discordance might occur, for example, when a competitor or predator invades or becomes extinct, a new resource enters the community, or the climate changes. If, however, environmental change within a community does not result in diversifying selection, but instead only results in an overall shift in the phenotypic optimum across the lineage, then this does not comprise niche discordance because selection does not favor increased phenotypic variance in this case and should thus not lead to phenotypic diversification. In the context of multiple communities, niche discordance occurs when a dispersing subpopulation colonizes a new community, and biotic or abiotic circumstances of the new community impose altered selection on niche-related traits, favoring niche expansion or a change in mean phenotype. In the terms of evolutionary landscapes, niche discordance reflects a shift in the individual selection surface such that new or broader regions of niche-trait space now experience high fitness. This scenario favors a broader occupation of the adaptive landscape by a lineage, either within a community, as populations diverge in a sympatric setting, or across communities, as allopatric populations diverge.

The concept of ecological opportunity is fundamentally coupled with the concept of the ecological niche. This connection is rooted in Darwin's view of ecology's determinative role in the formation of species and is made explicit in Gause's axiom that a species may persist within a community only if it differs sufficiently in ecological traits from other species (Hardin 1960). It follows that any addition of a new species to a community must involve filling a niche that is either unoccupied or vulnerable to usurpation. Thus, ecological opportunity requires the availability of a niche, so that a population possessing a particular phenotype previously absent from the community could inhabit that community (Box 1). While some previous references to ecological opportunity suggest that niche availability alone is sufficient to constitute ecological opportunity, this is incorrect because niche availability need not entail processes that can drive lineage divergence. Phenotypic diversification of a lineage is facilitated by niche discordance, where diversifying selection favors increased variance of niche-related traits (Box 2). For example, a marine stickleback population that colonizes an inland lake will likely experience niche discordance, with divergent selection on armor plates (favoring reduction in armor in freshwater) owing to the distinctly different predator community in the new habitat (Barrett et al. 2008; Colosimo et al. 2005).

With this framework, we offer a precise, working definition of ecological opportunity: *ecological opportunity is a prospective, lineage-specific characteristic of an environment that contains both niche availability, allowing a population to persist in the environment, and niche discordance, causing diversifying selection within the lineage*.

Our definition of ecological opportunity shares features with previous characterizations, especially with regard to niche availability, which is often a central element of the concept's antecedents (Lack 1947; Simpson 1944). Niche discordance, however, is often only implied, with phrases such as “few competitors” or “wealth of resources” to refer to changed ecological constraints. In some prior studies, “ecological opportunity” has been used interchangeably with “niche availability;” although many authors have also explicitly considered selection on niche-related traits in discussions of ecological opportunity (e.g., Losos 2010; Schluter 2000; Yoder et al. 2010). Our framework also differs from most treatments in that, rather than a primary or exclusive focus on resource competition, we emphasize that ecological opportunity occurs in the context of full communities and that ecological and evolutionary dynamics under conditions of ecological opportunity (e.g., speciation, adaptive radiation) are governed through interactions with resources, competitors, predators, mutualists, and the full array of biotic and abiotic circumstances of a community.

**Figure 2:**
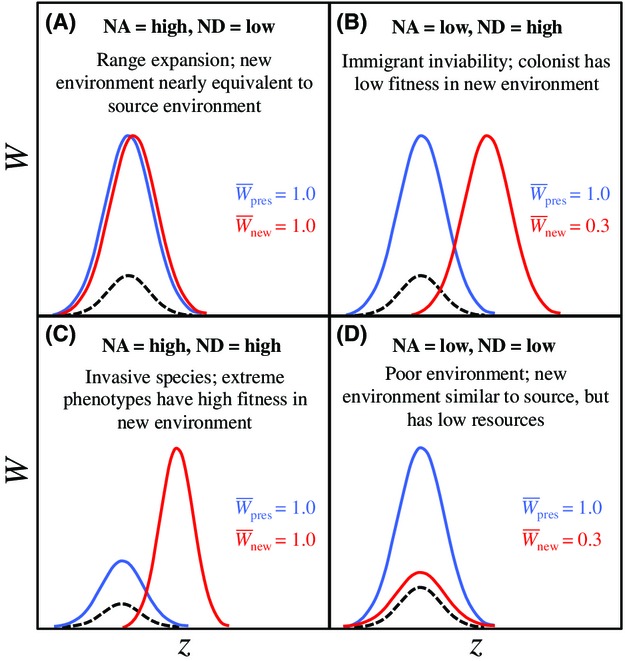
Example individual selection surfaces illustrating possible combinations of niche availability and niche discordance for scenarios involving an ancestral population (individual selection surface in blue) and a dispersing colonist population (individual selection surface in red). The dashed black curve in each panel represents the initial phenotype distribution of the colonist population (equal to the ancestral distribution). For each hypothetical example, we provide a plausible biological scenario and the relative values of mean population fitness for the phenotype already present in the community (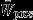) and the new phenotype previously absent (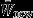). (A) The two populations have equivalent high values of mean population fitness (high niche availability) and nearly identical selection surfaces (low niche discordance). (B) Mean population fitness is initially much lower in the colonist population (low niche availability), with strong, divergent selection across populations (high niche discordance). (C) The two populations have equivalent high values of mean population fitness (high niche availability), and strongly divergent selection surfaces (high niche discordance). (D) Mean population fitness is much lower in the colonist population (low niche availability), and the selection surfaces are virtually identical (low niche discordance).

We suspect that understanding the relationships and interactions between niche availability and niche discordance in the wild will allow insights into ecological opportunity's role in generating biological diversity. However, we currently have little knowledge on this topic because no prior framework for ecological opportunity explicitly delineated the importance of these two elements. One might anticipate a negative correlation between niche availability and niche discordance, as a colonist population with high niche availability might typically already reside near a fitness peak and thus experience little niche discordance because the similar fitness surfaces across habitats impart little or no diversifying selection (Fig.2A). Moreover, cases of especially strong niche discordance might confer low niche availability owing to the absence or rarity of traits that would experience high fitness in the new environment (Fig.2B). However, this expectation could be naïve, as niche availability and niche discordance can simultaneously exist at either high or low levels (Fig.2C, D). For instance, a colonist population could experience no reduction in population growth rate (e.g., equivalent mean population fitness) and yet experience strong diversifying selection, such as steep directional selection toward a new, higher fitness peak, as is perhaps exemplified in some invasive species (Sultan et al. 2013; see Fig.2C). Alternatively, a colonist population might experience a strong reduction in population growth rate (low niche availability) even though the shape of the selection surface did not change (low niche discordance) owing to lower resource levels (Fig.2D). To better understand how ecological opportunity arises, and how it can drive adaptive diversification, we need further theoretical and empirical examination of the scope of interactions between niche availability and niche discordance.

**Figure 3:**
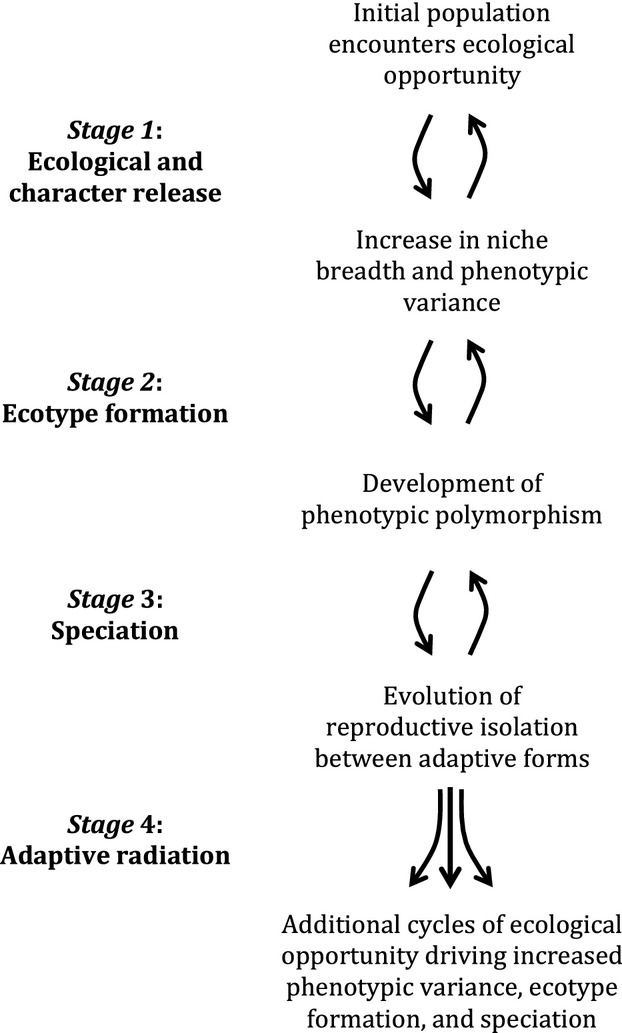
Dynamics of diversification in the presence of ecological opportunity. Bidirectional arrows emphasize that diversification need not be a ratchet-like, inevitable progression. Rather, divergence may remain in an arrested state of dynamic equilibrium, and accrued divergence may be lost to hybridization at any stage prior to evolution of irreversible reproductive barriers.

Diversification under the influence of ecological opportunity is expected to progress in a more or less generalized way that we frame as a series of four “stages” (Fig.3). In Box 3, we explore in detail the temporal process of lineage diversification under conditions of ecological opportunity across different spatial contexts, including delineating conditions that do, and do not, constitute ecological opportunity, and circumstances in which initial speciation enhances opportunity for additional rounds of speciation.

**Box 3. Process of Diversification Under Conditions of Ecological Opportunity**

Ecological opportunity can facilitate diversification through four general “stages” (Fig.3). However, advancement through each stage is not inevitable, and lineage diversification will often not proceed fully through all four stages, and may reverse, especially in its early stages (Nosil et al. 2009).

**Stage 1: Ecological and Character Release**

The initial expectation for a lineage experiencing ecological opportunity is an increase in phenotypic variance in traits associated with niche expansion or divergence (Nosil and Reimchen 2005; Parent and Crespi 2009). Although increased phenotypic variance may, at least originally, derive from phenotypic plasticity, we focus on evolutionary responses to ecological opportunity, which could include evolutionary changes in plasticity. Phenotypic plasticity may promote successful colonization of new environments by shifting the population mean phenotype in a manner that increases niche availability (Pfennig and Pfennig 2010; West-Eberhard 2003; Yeh and Price 2004); however, as long as niche discordance occurs, selection favors even greater phenotypic variance than initial plasticity provides.

**Figure 5:**
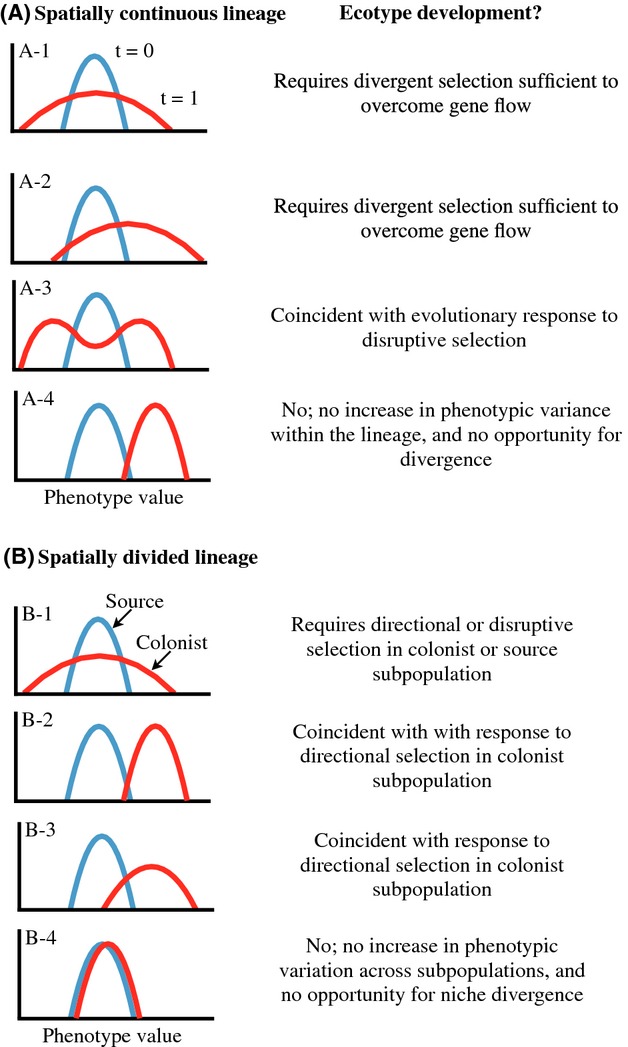
Processes that may operate during Stage 1 of diversification under ecological opportunity. For simplicity, we assume changes in phenotypic values are due to selection. (A) Processes operating in spatially continuous populations. Blue curve represents the phenotype frequency distribution of an initial population before encountering a change in selection due to niche discordance; red curve represents the population's initial evolutionary response to the new selection regime. (B) Processes operating in spatially divided populations. Blue curve represents a source population that does not encounter ecological opportunity; red curve represents a colonist population's initial evolutionary response to the (potentially) new selection regime. See text for further discussion.

In the presence of ecological opportunity, dynamics of evolutionary increases in phenotypic variance greatly depend on the degree of genetic intermixing among members of the population (Fig.5). First consider a spatially continuous, panmictic population that experiences an increase in ecological opportunity (Fig.5A) as may occur by a change in species composition within a community. Character release may involve an increase in phenotypic variance with no change in mean phenotype (A-1 in Fig.5), or an increase in phenotypic variance may be accompanied by a response to directional selection (A-2 in Fig.5). If disruptive selection is lacking, such populations may simply persist at higher phenotypic variance indefinitely (Bolnick et al. 2010). In contrast, character release in a population may occur in the form of strong disruptive selection in which an increase in phenotypic variance drives development of a bimodal distribution of phenotypes (A-3 in Fig.5) (Berner et al. 2009), as is likely when expanded ecological opportunity involves exploitation of discretely different niches, and adaptation entails significant functional tradeoffs between niches (Bolnick and Fitzpatrick 2007).

Alternatively, initial encounter with ecological opportunity may involve spatial division of a population initiated by dispersal of some individuals to a new location, resulting in little or no gene flow between source and colonist subpopulations (Fig.5). While the source subpopulation will usually remain unchanged, the colonizing subpopulation may experience divergent selection for niche expansion (B-1 in Fig.5) or niche shift (B-2, B-3 in Fig.5) in the new habitat, with concomitant evolutionary change in phenotype. Within the colonizing subpopulation, ecological opportunity in the new habitat precipitates an increase in phenotypic variance, change in mean phenotype, or both, and either response causes increased variance when considered across the full lineage (i.e., combined source and colonist subpopulations).

It is instructive to consider the nature of evolution in the absence of ecological opportunity. For a panmictic population, an adaptive shift in the phenotypic mean without increased phenotypic variance (A-4 in Fig.5), as may occur if phenotypic evolution tracks environmental change over time, does not reflect diversifying selection, and is not a result of ecological opportunity by our definition. Similarly, spatial division in the absence of divergent selection between source and colonist subpopulations (B-4 in Fig.5), as may occur when dispersing individuals occupy the same niche as the source population, will typically not result in any increased phenotypic variance. The lack of diversifying selection (i.e., no niche discordance) in both of these cases means that selection does not favor increased phenotypic variance within the lineage, and thus no ecological opportunity exists. Although these scenarios may involve considerable anagenetic change or allopatric speciation, even perhaps as a response to similar selection pressures (Langerhans and Riesch 2013), these outcomes do not derive from ecological opportunity, and do not entail new ecological diversity generated within the lineages.

**Stage 2: Ecotype Formation**

Divergent selection experienced under ecological opportunity can give rise to ecologically and phenotypically divergent intraspecific forms, which we refer to broadly as “ecotypes.” Ecotype formation can arise via genetically based polymorphism or phenotypic plasticity and can develop under any spatial context (Rundle and Nosil 2005). For a dispersing subpopulation that colonizes an ecologically novel habitat, a response to directional selection on ecological traits in its new habitat is coincident with ecotype formation (B-2, B-3 in Fig.5). Within a spatially continuous population, development and maintenance of genetically divergent ecotypes by disruptive selection may occur despite gene flow between them (A-3 in Fig.5), but, in comparison with spatially isolated populations, conditions for ecotype formation and persistence are more restrictive (Bolnick and Nosil 2007; Nosil 2008; Rueffler et al. 2006). Nonetheless, development and maintenance of ecotypes with gene flow may occur frequently (Eroukhmanoff et al. 2009; Smith and Skulason 1996; Storfer and Sih 1998), especially under conditions of an abrupt spatial discontinuity in ecological environments because intermediate phenotypes have low fitness (Berner et al. 2009; Rueffler et al. 2006). On the other hand, phenotypic plasticity may readily evolve in sympatry as a response to disruptive selection without a constraining role for gene flow (Doughty and Reznick 2004; Dudley 2004; Martin and Pfennig 2010; West-Eberhard 1989).

**Stage 3: Speciation**

The central issue in understanding the transition from ecotypes to species is identification of processes that produce strong barriers to reproduction between ecotypes (Langerhans and Riesch 2013; Nosil 2012). Although formation of genetically, phenotypically, and ecologically divergent ecotypes represents a prerequisite for speciation arising from ecological opportunity, development of reproductive isolation between ecotypes is not inevitable (Hendry 2009; Nosil et al. 2009). Ecotypes may form in isolated populations, but fail to evolve barriers to mating (Magurran 1998; Nosil et al. 2009), and ecotypes (and even species) may develop substantial reproductive isolation under divergent selection, but collapse into panmixis when divergent selection is relaxed (De León et al. 2011; Seehausen et al. 2008). Generally, research into the varied causes of “ecological speciation” addresses this stage of diversification under ecological opportunity (Langerhans and Riesch 2013; Nosil 2012).

Several lines of evidence suggest that divergent natural selection facilitates speciation (Hendry et al. 2007; Rundle and Nosil 2005; Schluter 2009). Two quantitative features of divergent selection that increase the likelihood of reproductive isolation are total strength of selection and selection on multiple independent traits (Nosil et al. 2009). Both strong and multifarious divergent selection may often be characteristic of higher levels of ecological opportunity. Greater levels of niche availability and niche discordance increase opportunities for niche shifts that entail large phenotypic change owing to the broader range of ecological space that is vulnerable to invasion and exploitation, whether niche occupancy occurs through dispersal or divergence under gene flow. High levels of niche discordance for multiple traits can facilitate evolution along multiple axes; although some iconic adaptive radiations resemble multidimensional divergence during speciation, such as the Hawaiian silversword alliance (Baldwin and Sanderson 1998), others appear to have diversified primarily along single niche axes, like diet composition (beak morphology) in Darwin's finches (Grant and Grant 2008).

**Stage 4: Adaptive Radiation**

Development of reproductive isolation between diverging populations can serve as a catalyst for further divergence and speciation. For instance, reduction of genetic exchange during divergence can increase the rate of niche adaptation (Storfer and Sih 1998; Garant et al. 2006; Bolnick and Nosil 2007; but see Seehausen 2004; Givnish 2010 for discussion of hybridization's potential to facilitate divergence and radiation). Moreover, because speciation mitigates migration load, speciation may facilitate future diversification of the now independently evolving species, allowing them to more freely explore the adaptive landscape.

Evolution of reproductive isolation also sets the stage for ecological and reproductive character displacement upon secondary contact (Taper and Case 1985), a process that can contribute to additional diversification under ecological opportunity. Character displacement may manifest as divergent natural selection within a species when the species experiences secondary contact with a sister species in some areas of the species' range but not others, and this divergent natural selection may initiate a new cycle of speciation, especially when divergence includes reproductive traits (Hoskin and Higgie 2010; Rice and Pfennig 2010).

## Extensive Ecological Opportunity in Depauperate Communities

As many authors have noted, the magnitude of ecological opportunity may increase in more depauperate communities – those communities with low species richness but sufficient resources to support additional species. Such conditions often characterize new habitats, including oceanic islands, postglacial lakes, ecologically novel habitats, and communities made depauperate by multispecies extinction events (Jablonski 2005). Ecological opportunity in these communities may be especially high because the relative paucity of negative interspecific interactions enhances both niche availability and niche discordance. In the absence of predation and competition, niche models suggest a population with any new phenotype has a high probability of establishing itself, provided it has the ability to survive and reproduce under the abiotic and resource supply conditions of the community (Chase and Leibold 2003). In a depauperate community, many configurations of a phenotype's ZNGI and impact vectors will allow establishment of the phenotype in the community, even for species initially poorly adapted to conditions of the community (Leibold 1998). Moreover, with few strong ecological constraints, substantial scope exists for changes in configuration of a phenotype's ZNGI and impact vectors (i.e., niche evolution) without risk of extinction from the community, suggesting high niche discordance in depauperate communities. Under these conditions, adaptive diversification through postcolonization evolutionary niche shifts or in situ diversification should be common. Substantial niche availability for so many new phenotypes, coupled with high niche discordance, may be characteristic only of highly depauperate communities, suggesting that these communities may display the greatest levels of ecological opportunity and highest per-lineage rates of adaptive diversification.

Extensive evidence from varied perspectives supports the view that depauperate communities provide centers of adaptive diversification arising from ecological opportunity. At the largest scales, elevated rates of ecological diversification following mass extinction events suggest both that extinction gives rise to greater niche availability and niche discordance and that a lack of ecological opportunity constrains diversification during the long intervals between extinction events. Extinction at the Triassic–Jurassic boundary is associated with increased ecological diversification of dinosaurs (Langer et al. 2010), and the Cretaceous–Paleogene mass extinction event that caused the demise of nonavian dinosaurs was followed by rapid and substantial ecological diversification of mammals (Smith et al. 2010).

Phylogenetic studies, and particularly those allowing inference of phenotypic evolution during community assembly, provide support for abundant ecological opportunity early in diversification of a clade when communities may be depauperate with respect to ecologically similar species (Cavender-Bares et al. 2009; Emerson and Gillespie 2008; Gillespie 2004). For example, in the evolutionary assembly of *Desmognathus* salamander communities in eastern North America, ecophenotypic evolution was concentrated within early stages of the radiation, followed by an extended period of species diversification with little ecophenotypic change (Kozak et al. 2005). In a somewhat related phylogenetic approach, several studies report the highest rates of lineage accumulation early in some radiations (Burbrink and Pyron 2010; Phillimore and Price 2008), a pattern consistent with high initial levels of ecological opportunity, followed by declining levels as ecological space fills. Although suggestive, caution is warranted when using phylogenetic studies alone to infer complex evolutionary mechanisms (Losos 2011).

Ideally, mechanisms by which ecological opportunity shapes development of biological diversity would be evaluated experimentally, and experimental diversification studies in microbes offer substantial insight into the action of ecological opportunity in low-diversity communities. In their landmark study, Rainey and Travisano (1998) demonstrated that, when introduced into static broth media, *Pseudomonas fluorescens* predictably diversifies into three primary ecophenotypic forms, the ancestral broth-adapted smooth form, the wrinkly spreader which forms a surface mat that allows it to capitalize on surface oxygen, and the fuzzy spreader that occupies the anoxic bottom region. This divergent evolution is driven by competition for resources and the associated fitness trade-offs arising from niche adaptation. Evolution of ecophenotypic forms is fully dependent on the ecological opportunity provided by a static medium, as no diversification occurs if the environment is made ecologically homogeneous by continual stirring. Subsequent studies found that increased ecological opportunity created by greater diversity of carbon substrates caused evolution of higher ecophenotypic diversity (Barrett et al. 2005), and conversely, that ecological diversification of *P. fluorescens* is progressively more restricted as ecological opportunity is reduced by the presence of one to four resident competitors (Brockhurst et al. 2007). Collectively, microbial evolution studies have experimentally verified fundamental mechanisms by which ecological opportunity causes adaptive diversification (Kassen 2009).

Replicate adaptive radiations in the wild also yield evidence for high levels of ecological opportunity in depauperate communities, and moreover, point to a strong element of determinism in the action of ecological opportunity (Baldwin 2007; Gillespie 2004; Losos 2010). These radiations demonstrate that similar habitats, comprising similar arrays of niches, yield similar patterns of ecological diversification, and as Gillespie (2004) stated, such patterns suggest “universal principles may underlie the process of community assembly.” The Bahamas mosquitofish, *Gambusia hubbsi*, for example, has repeatedly and independently evolved similar ecotypes across geologically young and biologically depauperate blue holes on Andros Island in response to the predatory environment (Langerhans 2009; Langerhans et al. 2007; Riesch et al. 2013). Replicate adaptive radiations that are themselves replicated across multiple distinct clades in response to the same ecological factors provide particularly strong evidence that diversification is driven by ecological opportunity in a more or less deterministic process. Fishes of geologically young postglacial lakes in the Northern Hemisphere provide an example. Two phenotypic forms of European whitefish, *Coregonus lavaretus*, often occur in northern European lakes, and these pelagic-feeding and benthic-feeding forms are associated with distinct niches. Microsatellite analyses indicate that species pairs most likely evolved independently across multiple lakes (Østbye et al. 2006). Similar patterns of independent parallel evolution of benthic and pelagic morphs are observed in other salmonids, including North American whitefish, *C*. *clupeaformis* (Landry et al. 2007), char (Snorrason et al. 1994), and salmon (Wood and Foote 1996), and in the unrelated threespine stickleback (Taylor and McPhail 2000). Of course, replicate radiations in similar environments are not inevitable (Losos 2010), but the several remarkable examples across a diversity of taxa underscore the manifest influence of ecological opportunity in shaping the form of biological diversity in low-diversity communities.

## Uncertain Role of Ecological Opportunity in Species-Rich Communities

The extent and character of ecological opportunity in species-rich communities is far from clear. Although ecological opportunity is often assumed to decline as communities become more species rich, recent critical evaluation of this assumption suggests unequivocal support is lacking (Benton and Emerson 2007; Losos 2010). On one hand, accrual of species in a community is expected to reduce ecological opportunity as accumulation of species constrains niche availability and niche discordance, an expectation supported by niche models and various sources of empirical evidence (Kassen 2009; Kennedy et al. 2002; Phillimore and Price 2008). Alternative perspectives, on the other hand, indicate ecological opportunity persists and may increase in species-rich communities (Mittelbach et al. 2007; Schemske 2009). This largely results from species interactions, even negative interactions, which may enhance both niche availability (Holt et al. 1994) and niche discordance (Schemske 2009), creating conditions in which diversity itself facilitates speciation (Armbruster and Muchhala 2009; Losos and Mahler 2010). Thus, increasing species richness may fill previously available niches, but create new ones at the same time.

Ecological models of community assembly offer support for a reduction in ecological opportunity as communities accumulate species (Grover 1994; Leibold 1998; McPeek 2012). These analyses suggest that although the number of unfilled niches may remain high in species-rich communities, niche availability declines in the sense that available niches become more restrictive with respect to traits required for a new phenotype to become established and that niche discordance is constrained by narrower niche breadth. For example, a simple model community with a single resource allows only one consumer species to exist, but the community is open to invasion by a predator species, and this addition of a predator allows a second consumer species to invade and coexist stably with the predator and initial consumer species (Holt et al. 1994; Leibold 1996). Further species additions are also possible in the community, suggesting that niche space remains available, but as species accumulate in communities, traits of new species must meet ever more stringent phenotypic criteria (Grover 1994; Leibold 1998; McPeek 2012). This narrowing window of niche availability for each additional species implies limited niche discordance due to ecologically constrained postcolonization niche evolution, and thus yields more restricted ecological opportunity.

Although community theory suggests species-rich communities harbor reduced ecological opportunity, natural communities are typically more complex than those captured in community models. Most prominently perhaps, prevalence and strength of coevolutionary dynamics may increase with diversity, leading to elevated rates of niche evolution among members of species-rich communities (Losos 2010; Mittelbach et al. 2007). Schemske (2009) compellingly argued that latitudinal differences in relative importance of abiotic versus coevolutionary drivers of adaptation may contribute significantly to the much higher diversity of tropical communities compared to those of temperate regions. In abiotically benign tropical communities, continuous reciprocal adaptation in a web of coevolutionary interactions ensures that adaptive niche evolution is ongoing on an ever-fluid adaptive landscape. In temperate communities, by contrast, adaptation to harsh but predictable abiotic conditions primarily drives niche evolution to an optimal phenotype, a stationary adaptive peak. A colonizing subpopulation may successfully invade a species-rich community because its phenotype is already well adapted to the community (high niche availability), but promptly diverge from the source population because niche evolution is ongoing among species (high niche discordance), driving divergence and speciation.

Additional mechanisms may also operate to create ecological opportunity in species-rich communities. Elevated rates of coevolution in species-rich communities create more opportunities for cospeciation and evolution of more specialized phenotypes, essentially creating niches by more finely dividing ecological space (Armbruster and Muchhala 2009). Additionally, it may be wrong to assume that niche shifts are more difficult in species-rich communities than in depauperate communities. For example, species-rich communities may generate a higher proportion of positive species interactions that drive niche evolution (Kawakita et al. 2010) or may have shorter average phenotypic distance between fitness peaks, allowing niche shifts with comparatively little phenotypic change (Munday et al. 2004). Clearly, much remains to be learned about ecological opportunity's role in the buildup of biological diversity in species-rich communities. We anticipate investigations of diversification in species-rich communities will provide new insight into the operation and importance of ecological opportunity in generating biological diversity. One challenge will be to reconcile, through theory and empiricism, the evidence for exhaustion of ecological opportunity with increasing species richness in simple model communities with seemingly high levels available in species-rich communities.

## Ecological Opportunity in Human-Altered Environments

While further theoretical work is needed, ecological opportunity should generally increase following large, rapid, and multifarious environmental shifts – like the changes that commonly result from human activities. This suggests that the strong and widespread environmental impacts of humans on a diverse array of biotic and abiotic factors may not only precipitate earth's sixth mass extinction event (Barnosky et al. 2011; Dirzo and Raven 2003; Leakey and Lewin 1992), but could also foster high levels of diversification in organisms for which environmental change creates substantial ecological opportunity. Human-induced rapid environmental change (HIREC) can cause a variety of changes to ecological communities that can influence ecological opportunity, such as:

1. Novel habitats (e.g., habitat modification, human structures)
2. Novel resources (e.g., agriculture, garbage)
3. Novel competitors (e.g., species introductions, range expansion)
4. Novel enemies (e.g., humans, introduction of predators/parasites/diseases)
5. Novel abiotic stressors (e.g., pollutants, climate)
6. Novel background environments (e.g., light/color, sound)
7. More depauperate communities (e.g., extinctions, local extirpations)

Not only may HIREC dramatically alter environmental conditions that affect ecological opportunity, but it may also modify factors that influence population responses to ecological opportunity. HIREC likely frequently alters the spatiotemporal structure of ecological opportunity, for instance by changing connectedness among populations (e.g., habitat fragmentation) or temporal variation in selection (e.g., altered seasonality through climate change). Moreover, HIREC can even affect diversification potential of populations, for example, by altering gene flow via translocation of organisms, resulting in genetic admixture (including hybridization) and altered responses to selection (Ellstrand and Schierenbeck 2000; Kolbe et al. 2008; Lavergne and Molofsky 2007; Nolte et al. 2005; Rieseberg et al. 1999).

Previous work has documented extensive effects of HIREC on community structure, phenotypic change, and rapid evolution (Hendry et al. 2008; Palumbi 2001; Scheffer et al. 2001; Sih et al. 2011; Vitousek et al. 1997). Thus, the environmental changes wrought by humans can clearly have major ecological and evolutionary consequences; but might these environmental changes generate both extensive ecological opportunity and adaptive diversification? Because many of the ways that humans alter the environment should result in new niche availability and niche discordance, we suggest that HIREC likely increases ecological opportunity in many cases. However, considering that until recently little work had investigated the potential diversifying force of HIREC, we currently have inadequate data to assess whether HIREC will eventually produce more extinction than diversification or vice versa.

The idea that HIREC may drive widespread patterns of diversification may seem counter to the ample evidence for biotic homogenization, the increased genetic, taxonomic, or functional similarity of biotas over time resulting from species extinctions and invasions (McKinney and Lockwood 1999; Olden 2008; Rahel 2000). But perhaps the occurrence of biotic homogenization indicates that human activities often create similar types of new niche availability within altered communities. If so, some of the new niche availability may most rapidly be colonized by species with high dispersal abilities that experience little niche discordance because their mean phenotypes already reside near the newly created adaptive peaks in human-altered communities (e.g., urban exploiters, invasive species). Meanwhile, occupancy of additional niche availability created by HIREC, for which most residents or colonizers experience strong niche discordance, may require longer time intervals for establishment and adaptation. Moreover, the many documented cases of rapid phenotypic shifts subsequent to anthropogenic environmental impacts appear to represent repeated responses to human-created ecological opportunity. If human-modified environments usually generate similar types of new ecological opportunity, and various species adapt to these repeated instances of new adaptive peaks, then this could result in functional homogenization across many localities through a process of contemporary adaptation. Indeed, many species may currently be in the process of diverging between subpopulations adapted to human-altered environments and ancestral subpopulations less impacted by human activities (or affected by different human impacts). Whether HIREC may often lead to speciation remains an open question, but speciation certainly seems ongoing in a number of cases (Filchak et al. 2000; Hendry et al. 2000, 2007; Schwarz et al. 2005), and future work could examine the frequency of human-induced differentiation in traits closely linked to reproductive isolation such as breeding/flowering time, breeding location, genitalia, or mating cues or preferences (Feder et al. 1994; Heinen-Kay et al. 2014; Hendry et al. 2000; McNeilly and Antonovics^1968^). We know that divergent natural selection often drives the evolution of reproductive isolation (Langerhans and Riesch 2013; Nosil 2012; Schluter 2009), we now need focused investigation of how HIREC might generate ecological opportunity and facilitate speciation.

## Spatiotemporal Structure of Ecological Opportunity

Dynamics of ecologically mediated diversification depend critically on the spatial context in which diversification unfolds (Doebeli and Dieckmann 2003; Emerson and Gillespie 2008; Urban 2011). The spatial structure of ecological opportunity defines the spatial distribution of form, direction, and intensity of selection, and prescribes the scope of spatial opportunities for speciation, primarily through impacts on gene flow (Kisel and Barraclough 2010). Ecological opportunity may also change temporally in form, intensity, and direction of divergent selection, and such fluctuations impact development and maintenance of intraspecific divergence and probability of speciation (Seehausen et al. 2008).

Although niche availability and niche discordance are necessary for diversification under ecological opportunity, these will result in speciation and adaptive radiation only under appropriate spatial and temporal conditions. Illustrative of this point is Lack's (1947) insight into the role of spatial structure in accounting for both the pronounced radiation of Darwin's finches among islands of the Galapagos archipelago where at least 13 species have formed and multiple species coexist on individual islands, and the complete absence of species diversification in the closely related Cocos finch (*Pinaroloxias inornata*) inhabiting the isolated and solitary island of Cocos. Lack (1947, p. 132) reasoned that “despite the length of time for which it has been there, despite the variety of foods and habitats which Cocos provides…there is still only one species of Darwin's finch … [b]ut Cocos is a single island, not an archipelago, and so provides no opportunity for the differentiation of forms in geographical isolation.” Cocos Island offers abundant niche availability and niche discordance, but without any spatial mechanism for genetic divergence. The Cocos finch has apparently increased phenotypic variance primarily through plasticity of feeding behaviors via learning rather than through evolution of ecotypes or speciation (Werner and Sherry 1987). Existence of multiple islands in the Galapagos, however, provides the possibility for dispersing individuals to adaptively diverge from the source population unimpeded by gene flow. Lack's explanation has stood the test of time (Grant and Grant 2008), and archipelagos in general provide spatial opportunity for divergence and speciation in birds (Kisel and Barraclough 2010). Temporal processes are implicit in Lack's explanation, first because ecological differences in allopatry must be sufficiently stable over time that divergence occurs and is maintained, and second because divergence in isolation must be sustained for a sufficient duration that accumulated phenotypic and genetic differences are not lost to hybridization upon secondary contact.

Spatial distribution of ecological opportunity impacts diversification by shaping patterns of gene flow between subpopulations, which in turn establishes dynamics of response to divergent selection and likelihood of speciation. Divergent selection may occur between geographically isolated populations at one extreme, or among spatially intermixed mosaics at the other, with intermediate spatial structures formed by spatially continuous populations distributed along gradual or abrupt ecological gradients. Dynamics of divergence and speciation differ across this spectrum of geographic arrangements (Bolnick and Fitzpatrick 2007; Coyne and Orr 2004). Speciation may be most likely when divergent selection acts between spatially isolated populations because divergence and speciation can advance unfettered by homogenizing effects of genetic recombination between diverging groups (Sobel et al. 2009), although under some conditions modest levels of gene flow may hasten speciation by, for example, causing selection on traits that promote reproductive isolation (Garant et al. 2006). While allopatric speciation can occur by nonecological mechanisms, theory and empirical evidence suggest that ecological opportunity plays a prominent role in many, perhaps most, cases (Schluter 2009; Langerhans and Riesch 2013; but see Rundell and Price 2009). Spatial ecological gradients and discontinuities also may produce divergent selection that leads to speciation, but strong divergent selection may be required to overcome genetic mixing across the gradient or discontinuity (Via 2009). Nonetheless, both theory (Doebeli and Dieckmann 2003) and the many empirical examples of divergence in the face of gene flow (Bernatchez et al. 2010; Nosil 2008) suggest that phenotypic divergence is not only possible, but common. It is less clear, however, that speciation is also common under these conditions (Berner et al. 2009; Nosil et al. 2009). In the absence of additional mechanisms driving completion of speciation, such as reinforcement, sexual selection, or genetic opportunities for speciation (Feder and Nosil 2009; Hoffman and Rieseberg 2008; Ritchie 2007), divergent ecotypes might persist indefinitely under a selection-migration balance. Advances in empirical assessment of gene flow during speciation will provide insight into the frequency and mechanisms of speciation in the face of gene flow (Papadopulos et al. 2011). At the finest spatial scales of ecological heterogeneity, disruptive selection can drive population divergence and speciation (Rueffler et al. 2006). Although possible, speciation in the absence of some level of spatial opportunity for divergence may be rare, perhaps exceedingly so (Bolnick and Fitzpatrick 2007; Mallet et al. 2009).

Temporal stability of ecological opportunity impacts the course of divergent evolution because multiple generations of divergent selection are usually required to generate ecological divergence and evolution of reproductive isolation (Hendry et al. 2007), and continued postspeciation selection may be required to sustain reproductive isolation (Seehausen et al. 2008). The time required for evolution of reproductive isolation under divergent ecological selection is likely shorter than for speciation under similar selection (Funk et al. 2006; Rundle and Nosil 2005), but even extraordinarily rapid ecological speciation requires greater than ten and often more than 100 generations (Hendry et al. 2007). Studies of selection in nature suggest temporal fluctuation in direction and intensity of selection is common (Hendry et al. 2009; Siepielski et al. 2009), and temporal fluctuation in selection across habitats may favor a single generalist phenotype rather than divergent habitat specialists (Sultan and Spencer 2002). Moreover, several studies demonstrate reversal of evolutionary divergence following environmental change (Seehausen et al. 2008). Finally, we note that temporal opportunity for speciation is also possible, as when divergent selection alters reproductive phenology (Savolainen et al. 2006).

## Diversification Potential

Ecological opportunity provides an environmental substrate conducive to diversification, but its evolutionary consequences depend on characteristics of populations that experience the environment. Building from Grant and Grant (2008), we use the term “diversification potential” to refer to those properties of a population that impact its potential to encounter ecological opportunity, and subsequently undergo divergent evolution and speciation in response to ecological opportunity. The concept of diversification potential has long been integral in discussions of ecological opportunity (Mayr 1963), and previous work reviewing topics such as traits associated with among-clade variation in speciation rates (Coyne and Orr 2004; Jablonski 2008) have generally indicated that diversification potential varies among populations, species, and clades. While undoubtedly difficult to measure, different organisms certainly differ in their probabilities of encountering ecological opportunity, evolving increased phenotypic variance, and undergoing speciation (Box 4). Diversification potential can involve traits that facilitate any stage of diversification under environmental conditions of ecological opportunity (Box 4), although historically researchers have largely centered on the potential for speciation rather than other stages.

**Box 4. Diversification Potential**

Diversification potential refers to the properties of a population that influence its potential to encounter and respond to ecological opportunity. While phenotypic and lineage diversification can occur in the absence of ecological opportunity, such as through genetic drift, nonecological sources of sexual selection, and differential responses to similar selection pressures (see Langerhans and Riesch^2013^), we more narrowly restrict *diversification potential* to refer specifically to the context of diversifying selection caused by ecological opportunity. Diversification potential comprises three components97:1276

- *E*: probability that ecological opportunity of a magnitude sufficient to potentially cause speciation is encountered by a population
- *V*: probability that phenotypic variance of ecological traits increases following the encounter with ecological opportunity
- *I*: probability that reproductive isolation evolves following increased phenotypic variance

*E*: A variety of factors impact the probability of encountering ecological opportunity, including dispersal, persistence, phenotypic variability, and niche construction. Higher dispersal frequency allows greater sampling of habitats within the dispersal range, and greater dispersal range increases the spatial scope of habitats encountered, and both affect probability of colonizing ecologically novel habitats (Kisel and Barraclough 2010; MacArthur and Wilson^1967^). Traits that facilitate persistence increase *E* because populations persisting though episodes of environmental change may take advantage of resources and habitats made available by changes in the community such as extinction of competitors and predators or altered resources (Archibald and Deutschmann^2001^; Asher et al. 2005; Benton 2010; Bernatchez et al. 2010). Population phenotypic variability enhances diversification potential because more variable populations may encounter a greater scope of niche availability in the environment, such as experiencing additional adaptive peaks far from the population mean. For example, enhanced phenotypic and genetic variation resulting from hybridization and introgression can increase the likelihood of expansion to new ecological settings and foster speciation (Givnish 2010; Grant and Grant 1996; Mallet 2007; Schwarz et al. 2005; Seehausen 2004). Other sources of population variability, including standing genetic variation (Schluter and Conte 2009) and phenotypic plasticity (Ghalambor et al. 2007; West-Eberhard 2003, 2005) can also increase niche availability. Finally, the abilities of organisms to construct, modify, and select aspects of their environment – niche construction – can influence *E* by creating new niche availability or niche discordance through such activities (Erwin 2008; Odling-Smee et al. 2003).

*V*: Population characteristics that increase *V* include any attribute that facilitates phenotypic evolution in response to natural selection. Magnitude and form of response to selection depends on many factors, including selection intensity, degree and form of genetic (co)variances of traits in a population, level of gene flow between diverging subpopulations, pleiotropic effects, and effective population size (Hartl and Clark 2006). Plasticity may enhance opportunities for adaptive evolution (Ghalambor et al. 2007; Pfennig et al. 2010; West-Eberhard 2003, 2005), and hybridization may simultaneously increase both phenotypic and genetic variance (Givnish 2010). Empirical studies of response to selection on a focal trait often find agreement with simple quantitative models (Galen 1996; Grant and Grant 1995), but genetic architecture and fitness tradeoffs among traits may add substantial complexity to a population's response to selection (Kirkpatrick 2009; Schluter 2000).

*I*: Considerable research has examined population characteristics that elevate the likelihood of speciation following a response to divergent selection (Givnish 2010; Nosil et al. 2009; Rundle and Nosil 2005). Three particular characteristics – limited dispersal, sexual selection, and genetic mechanisms –may be especially influential. Traits associated with restricted dispersal of individuals between diverging populations may give rise to evolution of reproductive isolation by reducing rates of recombination (Felsenstein 1981; Nosil 2008), and dispersal distance is closely associated with spatial scale of speciation across taxa on islands (Kisel and Barraclough 2010). In some cases, characteristics of newly colonized habitats select for reduced dispersal, as in the evolution of flightlessness in island birds (Livezey 1992, 2003; Slikas et al. 2002) and insects (Medeiros and Gillespie 2011). However, elevated dispersal may not always hinder progress toward speciation. Dispersal can impede speciation by elevating gene flow, but enhance the possibility of speciation by increasing encounters with ecological opportunity, suggesting highest rates of speciation may sometimes occur at intermediate levels of dispersal (Garant et al. 2006; Garb and Gillespie 2009). Dispersal may additionally induce completion of speciation by initiating the process of reinforcement (Servedio 2000; Servedio and Kirkpatrick 1997). Because sexual selection acts directly on traits involved in reproduction, it may advance completion of speciation, and may prove most effective in driving speciation when it occurs in concert with divergent natural selection (Coyne and Orr 2004; Ritchie 2007; but see Maan and Seehausen 2011). For example, adaptive divergence in body shape among Bahamas mosquitofish populations occupying different predator regimes causes premating reproductive isolation due to sexual selection acting on body shape (Langerhans and Makowicz 2013; Langerhans et al. 2007). Finally, genetic mechanisms that provide an avenue for overcoming recombination between diverging populations, such as one-allele mechanisms of assortative mating (Felsenstein 1981; Servedio and Noor 2003) and chromosomal inversions (Lowry and Willis 2010; Michel et al. 2010), also facilitate development of reproductive isolation under ecological opportunity.

One way to conceptualize the probability of speciation (*S*) in a population experiencing ecological opportunity over some time interval is to consider *S* as the product of three probabilities, *E*,*V*, and *I* (see Box 4) – the three components of diversification potential. Because *S *= *E *× *V *× *I*, a low value of either *E*,*V*, or *I* will greatly impede diversification even if other values are high. Thus, extensive adaptive radiations are likely characterized by high values of all three probabilities, as may be the case for well-studied radiations (Parent and Crespi 2009; Wagner et al. 2012). Some groups may fail to diversify because one probability is low, despite high values of the other two parameters. For example, spatially divided populations of Trinidadian guppies, *Poecilia reticulata* experience divergent natural selection imposed by alternative regimes of predation, and readily diverge in ecological traits, but do not evolve reproductive isolation (Magurran 1998). Although *E*,*V*, and *I* may each limit rates of diversification in specific lineages or ecological conditions, we generally have only idiosyncratic knowledge of how relative values of these probabilities vary across taxa and environments, and whether one probability overwhelmingly constrains diversification.

Historical discussion of ecological opportunity and adaptive radiation has often considered “key innovations,” traits that permit taxa to interact with the environment in novel ways and lead to increased rates of diversification (Losos 2010; Mayr 1963; Simpson 1944). Key innovations cause elevated diversification rates through increases in any combination of *E*,*V*, or *I*. For instance, the evolution of wings capable of flight in birds, bats, and pterosaurs may have dramatically increased *E*; the decoupling of pharyngeal and oral jaws in cichlid fishes may have greatly enhanced *V*; and complex communication structures in birds (syrinx) and frogs (ear papillae) may have strengthened *I*. Although their identification and study presents conceptual and empirical challenges (Donoghue 2005), novel traits linked with increased *E*,*V*, or *I* appear associated with many radiations, and may underlie the most dramatic radiations in the history of life.

## Conclusion

Ecological opportunity has formed the core of our ideas for the evolution and buildup of biological diversity since Darwin's description of the basic concept in *On the Origin of Species*, yet we have lacked a comprehensive framework for elucidating its scope, action, and consequences. Our goal has been to provide greater clarity to the concept of ecological opportunity by defining its fundamental elements, and we hope this exposition motivates more focused theoretical and empirical development. Much work remains. A central obstacle to a deeper understanding of the role of ecological opportunity in shaping the diversity of life is development of predictive approaches to its action. Despite the substantial conceptual importance of ecological opportunity, apparent from the concept's longstanding utility in evolutionary biology, it has so far lacked a predictive framework (Losos 2010). Such a framework may allow us to understand, for example, why adaptive radiations occur in some cases or habitats but not others. The obvious path to a predictive framework involves quantification and integration of niche availability, niche discordance, spatiotemporal structure of ecological opportunity, and diversification potential, all of which have been quantitatively examined *individually* in various ways (e.g., Kisel and Barraclough 2010; Nosil 2012; Schluter 2000; Schluter and Grant 1984). A stumbling block to unification of these elements, however, is the disparate approaches and metrics inherent in each component. Nonetheless, innovative methods for integration of components will likely be rewarded with significant new applications and insights that will illuminate details of how ecological opportunity shapes biological diversity.

Ecological opportunity lies at the intersection of community ecology and evolutionary biology, and advances in our understanding of adaptive diversification will benefit most from explicit synthesis of these disciplines. Are niche availability and niche discordance often positively or negatively associated in nature, and what conditions influence these associations? We need to understand how properties of ecological opportunity, its spatiotemporal structure, and diversification potential impact the scale of diversification. Do especially extensive or rapid radiations arise primarily from markedly high levels of ecological opportunity, or favorable spatiotemporal structure, or is elevated diversification potential most important? We need to elucidate broad patterns in the distribution, action, and magnitude of ecological opportunity. Of particular interest in this regard is determination of ways that ecological opportunity may differ quantitatively or qualitatively between depauperate and species-rich communities. Answers to this question will shed light on whether communities become saturated, or whether ecological opportunity is not limited by current diversity, or may in fact accelerate with increased diversity (Losos and Mahler 2010; Schemske 2009). Moreover, are ongoing human-caused environmental perturbations causing increased ecological opportunity, and potentially driving widespread ecological diversification? The fact that we do not yet know the answers to these important questions highlights just how much is yet to be learned about Darwin's fundamental driver of biological diversity.
